# Time to decide about risk-reducing mastectomy: A case series of *BRCA1/2 *gene mutation carriers

**DOI:** 10.1186/1472-6874-7-3

**Published:** 2007-03-06

**Authors:** Mary McCullum, Joan L Bottorff, Mary Kelly, Stephanie A Kieffer, Lynda G Balneaves

**Affiliations:** 1 Hereditary Cancer Program, BC Cancer Agency, Vancouver, BC, Canada; 2 School of Nursing, University of British Columbia, Vancouver, BC, Canada; 3 Faculty of Health and Social Development, University of British Columbia – Okanagan, Kelowna, BC, Canada; 4 Department of Medical Genetics, University of British Columbia, Vancouver, BC, Canada

## Abstract

**Background:**

The purpose of this research was to explore women's decision-making experiences related to the option of risk-reducing mastectomy (RM), using a case series of three women who are carriers of a *BRCA1/2 *gene mutation.

**Methods:**

Data was collected in a pilot study that assessed the response of women to an information booklet about RM and decision-making support strategies. A detailed analysis of three women's descriptions of their decision-making processes and outcomes was conducted.

**Results:**

All three women were carriers of a *BRCA1/2 *gene mutation and, although undecided, were leaning towards RM when initially assessed. Each woman reported a different RM decision outcome at last follow-up. Case #1 decided not to have RM, stating that RM was "too radical" and early detection methods were an effective strategy for dealing with breast cancer risk. Case #2 remained undecided about RM and, over time, she became less prepared to make a decision because she felt she did not have sufficient information about surgical effects. Case #3 had undergone RM by the time of her second follow-up interview and reported that she felt "a load off (her) mind now".

**Conclusion:**

RM decision making may shift over time and require decision support over an extended period.

## Background

As testing for *BRCA1/2 *gene mutations becomes more widely available as a clinical service, increasing numbers of women are being identified at high risk for breast and ovarian cancer. Female *BRCA1/2 *carriers are told they have an estimated lifetime risk of breast cancer between 50% and 85% [1]. Risk-reducing mastectomy (RM) is one option for breast cancer risk reduction that is offered to women who learn they are carriers of a *BRCA1/2 *gene mutation. Although reported interest in RM varies by clinic setting and country, up to half of women at high risk for breast cancer express either the intention to have RM or some uncertainty about this decision [2-4]. Our clinical observations indicate that with increased access to *BRCA1/2 *genetic testing, more high-risk women are considering RM and the majority of these women request additional information and support with this difficult decision.

An emerging body of research describes high-risk women's experiences and satisfaction with RM [5]. In one of the first long-term studies of the psychological impact of carrying a *BRCA1/2 *mutation and risk-reducing surgery, women reported adverse consequences of RM (i.e., changes in sexual function and body image), although the majority of the women believed the benefits of decreased fear outweighed the negative outcomes they experienced [6]. Another recent study found that half of women who had RM experienced ongoing physical problems up to 18 months following their surgeries [7]. Re-operations have also been reported as common following RM with implant reconstruction, usually for implant-related issues [8]. Bresser et al. and Metcalfe et al. explored satisfaction with RM and subsequent breast reconstruction with similar results; women were satisfied overall, with a significant number reporting post-operative complications [9,10]. Women's satisfaction with decision making specific to breast cancer risk-reducing strategies has been shown to be enhanced with the use of decision aids or a tailored decisional support system [11,12]. However, there are relatively few studies that have assessed high-risk women's actual decision-making processes related to breast cancer risk management.

Although high-risk women may receive clinical recommendations about prophylactic oophorectomy, chemoprevention, and breast screening, most clinicians are much less directive about RM and encourage women to make their own decisions. Because of the highly personal nature of this decision, most health care providers attempt to support women's decision making about RM rather than make recommendations for or against surgery. The decision-making process related to RM is complex because of the individual nature of the decision, and the implications that RM holds for women's body image are not well understood. Women experience uncertainty and ambivalence about RM [13,14] and health care providers may be unsure about how best to support women's RM decision-making. Among the many decisions faced by women at high risk for breast cancer, decision making related to RM is significant because asymptomatic women are considering an irreversible surgical procedure that has the potential to impact their physical and emotional well-being for the rest of their lives.

The purpose of this research was to explore women's decision-making experiences related to the option of RM, using a case series of three women who are carriers of a *BRCA1/2 *gene mutation. Three cases with different decision outcomes were selected to explore the complexity of the decision-making process, and the manner in which women deliberated over and revisited their decisions before resolving their treatment choices.

## Methods

Data were collected in a pilot study that assessed the response of women to a decision-making intervention related to RM. Because of the exploratory nature of the study, a mixed-methods approach was used that included standardized measures as well as open-ended interview questions to fully capture women's experiences.

The study explored high-risk women's decision-making processes in order to guide efforts to provide decision support about RM. Eligibility criteria for the pilot study included: 1) being at high risk for hereditary breast cancer; 2) requesting assistance from the provincial Hereditary Cancer Program with decision making about RM; 3) did not have breast cancer; 4) were over 18 years of age; and 5) spoke and read English. Ethics approval was granted by institutional ethical review boards and all participants gave informed consent.

Following baseline data collection, participants received a booklet describing RM developed by the research team. The booklet included available evidence related to expected benefits and potential risks associated with RM, and a worksheet to guide decision making. Women were offered a follow-up consultation either by telephone or in person with one of the co-investigators, a hereditary cancer nurse educator (MM). We contacted women by phone on two occasions following the consultation to collect data about their decision-making process. Questionnaires, developed by the investigative team, were administered to participants at baseline, and approximately two and six months after their consultation. The questionnaires measured self-perceived knowledge of breast cancer screening and RM, factual knowledge of breast cancer and RM, breast cancer worry, mood state, decision conflict, decision confidence, degree of satisfaction with the RM decision support booklet, as well as family cancer history. The measures that are reported on are defined below Table 1. The final telephone contact also included a semi-structured interview to review and verify women's decision-making experiences over time and to obtain additional data regarding factors that influenced their decision making. All telephone interviews were audio-taped and transcribed verbatim.

**Table 1:** Participants' knowledge and decision measures related to risk-reducing mastectomy (RM) at follow-up intervals

	**Case #1**			**Case #2**			**Case #3**		
**Measure**	**Baseline**	**1^st ^Follow-up**	**2^nd ^Follow-up**	**Baseline**	**1^st ^Follow-up**	**2^nd ^Follow-up**	**Baseline**	**1^st ^Follow-up**	**2^nd ^Follow-up**

Knowledge score^1^	6/10	8/10	9/10	9/10	9/10	9/10	7/10	7/10	7/10
Perceived level of knowledge of RM^2^	5/10	6/10	8/10	7/10	5/10	5/10	7/10	7/10	9/10
Perceived level of knowledge of breast screening^2^	4/10	7/10	8/10	7/10	5/10	7/10	8/10	8/10	6/10
Decision conflict^3^	2.42	2.58	2.58	1.0	1.83	3.33	2.16	1.83	2.33
Confidence with decision making^4^	n/a	9.2	8.2	n/a	8.0	5.0	n/a	9.5	9.5
Decision status^5^	Leaning to RM	Leaning to RM	Decided no RM	Leaning to RM	Leaning to RM	Undecided	Decided yes RM	Leaning to RM	Had RM

^1^Knowledge was measured using a series of ten true/false questions that were developed to assess women's knowledge about RM.

^2^Participants rated their perceived level of knowledge about RM and breast cancer screening on a ten point scale, where 1 is poor and 10 is excellent.

^3^Decision Conflict Scale [22,23] – A 12-item 5 point scale that assesses four areas of decision making: (uncertainty, feeling uninformed, clarity of values, and feeling unsupported). Scores may range from 1 (low decisional conflict) up to 5 (high decisional conflict).

^4^Confidence with Decision Making [24] – A 4-item 11 point scale that assesses level of confidence that advantages and disadvantages are understood, that a decision will be made, and of talking with health care providers about RM. Scores may range from 0 (not at all confident) to 10 (completely confident).

^5^Decision status [23]: Participants were asked to select the one of the following statements that best described their current thoughts about RM: You have decided to have prophylactic mastectomy; You are leaning towards having prophylactic mastectomy, but have not made a final decision; You have not decided one way or the other about prophylactic mastectomy; You are leaning towards not having prophylactic mastectomy, but have not made a final decision; You have decided not to have prophylactic mastectomy.

We selected three cases to represent different decision outcomes regarding RM among the women who tested positive for a *BRCA1/2 *gene mutation. Data from the questionnaires and interview transcripts were analyzed. Summative scores for study measures were calculated for each time interval (see Table 1) and content analysis of interview data was completed. The measures at the three time points were evaluated to identify individual trends in the decision-making process. These trends were then compared to the qualitative data and a synthesis was developed to provide a detailed narrative of each woman's decision-making trajectory. Minor changes have been made to the details of each case to maintain participants' confidentiality (e.g. we do not identify whether they had a *BRCA1 *or *BRCA2 *gene mutation, and have modified their family histories) and permission to report each case was obtained.

## Results

A description of each woman's decision-making experiences is presented to reflect their narratives and the context in which their decisions were embedded.

### Case #1

This 40-year-old healthy woman began to think about RM after she received a positive *BRCA *genetic test result. She had attended genetic counselling several months earlier with her sister, who also received the same result. Although RM was presented as one of the options to consider during her genetic counselling sessions, it was at her first high-risk screening clinic appointment, several weeks after receiving her test result, that she requested more information about RM and was given the RM booklet.

Breast cancer was not an unfamiliar event for this woman. Her mother, one aunt, two maternal great-aunts and two of her mother's cousins had all been diagnosed with breast cancer, and several friends had received cancer diagnoses at young ages. After her mother's breast cancer diagnosis eight years earlier, this woman stopped doing regular breast self-examinations, because she felt too anxious about the likelihood of finding a lump. She admitted that worry about breast cancer strongly affected her ability to perform daily activities even though none of her close female relatives affected by breast cancer had died of their disease. At baseline, she perceived her knowledge of breast cancer screening and RM to be low, and she reported a moderate level of decision conflict (see Table 1).

In her follow-up consultation, two months after receipt of the RM booklet, she stated that when she first learned about RM, she felt that she "didn't have a choice," and was, therefore, leaning towards having RM. After "lots of thinking about it" and searching out information, she learned that surgical removal of her ovaries would "free her" from the worry of ovarian cancer, which is associated with carrying a *BRCA *gene mutation, and also reduce her risk of breast cancer. She decided that undergoing an oophorectomy (and hysterectomy) was the "first step, the smartest step" and represented an option that was "less radical" and "made much more sense" for her than having her breasts removed. Because this woman had a history of painful menstrual cycles and already had children, she felt that she "did not need" her uterus and ovaries anymore; furthermore, she perceived the hysterectomy to be a decision with "internal" effects only.

Compared with risk-reducing oophorectomy and associated hysterectomy, thinking about RM resulted in far more unanswered questions for this woman, especially with regard to details of the surgery and breast reconstruction. She referred to RM as a "stage two option" that might be taken in the future, whereas she viewed herself at "stage one". The woman reflected on her approach to making a decision about RM and said, "I think I have to do it in a step process to know that this is absolutely what I have to do." At this time, she described regular breast screening as a pro-active step she could take to protect herself, and wanted to find out how screening would affect her emotional health over time.

Three months after the consultation, during the first follow-up interview, she indicated she was still leaning towards having RM, but had not made a final decision. Her perceived knowledge of RM and breast screening had increased slightly by this time, and her decision conflict remained stable, but she scored extremely high on the decision confidence scale. Six months later, at the second follow-up interview, this woman talked convincingly about her positive experience with oophorectomy/hysterectomy some months earlier, and reported that she had further modified her decision about RM. Confident that removing her ovaries had "covered all her bases" in terms of preventing cancer, she had decided not to have RM. At this time, her perceived knowledge levels had again increased slightly, her decision conflict was unchanged, and her decision confidence remained high.

Reflecting on the shift in her decision making over time, this woman stated that her initial reaction was made "without really thinking it through" and that she now realized that RM was "too radical" and that unlike ovarian cancer, early detection methods were an effective strategy for dealing with breast cancer risk. She reported that breast self-exam was easier after her oophorectomy without as much estrogen circulating in her body, and she believed she would be able to detect a significant change in her breasts, if one occurred, or that it would be found by the high-risk breast cancer screening program in which she was now participating.

When asked how the RM booklet had influenced her decision, she described the booklet as something that "helped her a lot" in terms of organizing her thoughts, weighing the pros and cons of each option, preparing her to make a better decision, and ensuring that her time with doctors ran "more smoothly." However, she also expressed a desire for more information than was provided, especially in the form of example case studies. When asked what would be most helpful if she was to revisit her decision about RM in the future, she identified, as a priority, the opportunity to speak with women who had experienced RM firsthand.

Her reflection on decision making about RM illustrates her increasing uncertainty and the unexpected complexity of this decision:

I think that it's something that you really do have to think long and hard about. Really have to look at all the risk factors. You have to have all the information in front of you. Talk to your health care professional. Talk to your spouse or whoever is important. It's not something that you can just make a split decision and just, 'This is what I'm going to do,' cause that's what I did initially and it's probably a reaction that a lot of people have. 'That's it. I'm just going to have them off, to do this. I've made the decision'. It's not that easy of a decision. Something that you really have to think about because this, it's big. It's a big surgery.

### Case #2

This 49-year-old woman, who described herself as calm, secure and relaxed, found that her decision about whether to have RM became increasingly difficult over time. Genetic testing by a relative with both breast and ovarian cancer had identified a *BRCA *gene mutation in her family. She pursued genetic testing after her mother, who had recently been diagnosed with breast cancer, was found to have the same gene mutation. Four months after receiving her own positive result, this woman initiated a discussion about RM during her appointment at the high-risk screening clinic and was given the RM booklet. Her baseline scores showed that she perceived her knowledge about breast cancer screening and RM to be relatively high, and that she experienced no decision conflict (see Table 1). She stated that she leaned strongly towards having RM after she received her genetic test results and learned about the surgical option.

At her consultation, two months after receipt of the RM booklet, she expressed concerns about her tentative plan to have risk-reducing oophorectomy, and appeared more focused on this decision than on RM. She was particularly worried about the after-effects of "instant menopause" and raised issues about hormone replacement therapy, quality of life, and associated health risks. Her sister experienced an extremely difficult time after undergoing hysterectomy (including oophorectomy), and she worried that her quality of life might diminish as well. Although she thought she would "go ahead" with the surgery, she wanted to talk with other women who had already had the procedure.

Nevertheless, at this time she maintained she was still leaning towards having RM and was emphatic that she was not concerned about losing her breasts and her reproductive organs. She admitted, however, to feeling "too nervous" to look at online photographs of breastless women because they might be "too graphic". She raised questions about breast reconstruction options and procedures, and wanted to speak with women who could tell her about the experience firsthand. When she shared information about RM with her sisters and her mother, she said they were "shocked" that she would seriously consider such a "severe choice." One sister stated that she had heard horror stories from women who had gone through this surgery. The participant concluded that if she decided to have RM, she would not be able to tell her family about her decision until after the surgery.

At the first follow-up interview, she had already had her oophorectomy/hysterectomy and was still considering RM. Her decision conflict score related to RM was slightly higher than her baseline score, while her perceived knowledge levels had decreased (see Table 1). She maintained that she was still leaning towards a decision to undergo RM. Although she still had unanswered questions about the effects of the surgery, her high decision confidence score was indicative of her perceived ability to make a decision about RM.

However, four months later at the second follow-up interview, this woman described herself as "not decided one way or the other about RM," and reported that she was now less confident in her ability to make a decision, and less confident that she understood the risks. Study measures showed that her decision conflict score had increased again, while her decision confidence score had decreased, and her perceived knowledge level scores remained at moderate levels. She found that the decision became increasingly difficult as she gathered more information and developed new questions about breast reconstruction. She wondered about how her body would look without breasts and after reconstruction, the possibility of scar tissue build-up, the effect on her body if she elected to have larger breasts constructed, and the longevity of implants or reconstruction over time. She appeared to have realized that she had not gathered all the information she needed and she stated she might need to make time to re-read the RM booklet to help clarify some of the issues that had arisen for her.

One of the strongest factors influencing this woman's decision making about RM was her disappointment with the surgery she had to prevent ovarian cancer and reduce her risk of breast cancer. The severe symptoms she experienced afterwards, attributed to "the hormone battle" caused by the surgical removal of her ovaries, prompted her to begin using hormone replacement therapy (HRT), a decision that she questioned because of links between HRT and breast cancer. She realized that she did not have sufficient information about the implications of her first surgery and that there might have been other options she could have considered. She seemed determined to not let this happen again with other decisions, particularly RM. The inconsistent medical advice regarding RM (and also HRT) provided to her, her sister and her mother (each living in a different province) increased her confusion and indecision, and left her feeling angry and frustrated.

The fact that no one in her family had died from breast or ovarian cancer seemed to add to this woman's indecision about RM. Because her mother's breast cancer was diagnosed at an older age and she thought it was caused by long-term use of HRT, this woman did not currently perceive her own breast cancer risk to be extremely high, despite her genetic test results. She worried about how her body would look following breast removal and about scarring that would be left. Reflecting on the course of her decision making and the importance of information, she remarked:

It has gotten more and more difficult, [whereas] at the very beginning it just seemed like a no-brainer... I'm still not clear about how many surgeries and I'm still not, and when I say clear, it's not perfectly clear about the skin. I thought that I had the implants at the time of the surgery but it sounds like you don't. It sounds like you have to have the skin extenders in and then go back a second time for the implants. All that kind of stuff, it's not really clear to me.... I'm not complaining or anything like that. But I'm just saying it's a very difficult situation, there's just not enough information out there, long term.

She decided that she needed to go back and speak with the surgeon again. In the meantime, she was committed to participating in regular breast screening to provide the reassurance she needed while putting the decision about RM "on the back burner" Her indication that she was more inclined to withstand the surgery rather than risk ever being told she has cancer, suggests that this decision would not be put aside for the long term.

### Case #3

This 59-year-old woman had an extensive history of breast cancer on both sides of her large family, with two sisters, two aunts and four cousins having been diagnosed with the disease. Not surprisingly, worry about breast cancer affected her mood "a lot." Five months after she received positive *BRCA *gene test results, she felt ready to "take action" and received the RM booklet on the suggestion of her sister, who was aware of her plans to undergo both RM and risk-reducing oophorectomy. This woman stated that her initial decision was to have her breasts removed, and although she never completely changed her mind, post-operative complications from her oophorectomy created additional doubts and it became difficult for her to finalize a decision about RM. She wondered when, and if, she would be ready to undergo another surgery. At baseline, she perceived her knowledge of breast cancer screening and RM to be good and she reported a moderate level of decision conflict (see Table 1).

At her consultation, two months after receiving the RM booklet and a week after oophorectomy surgery, she stated, "I can't honestly say that I'm not scared...but I really feel in my heart that it's [RM] the right decision." At the first follow-up interview, she reported that the booklet helped her "a great deal" to organize her thoughts about this decision, consider the pros and cons of RM, and identify the questions she needed to ask. In interactions with family, friends and doctors, she sought opinions and gauged their reactions to assist her in finalizing her decision. Turning to health care professionals for advice, she found they would not weigh-in on the decision. She described how she repeatedly attempted to gain the personal opinion of health care providers and how a "slip" by one oncologist was interpreted as approval to go forward with surgery. She found this to be reassuring. The breast cancer experiences of two sisters, one of whom died of her cancer, were also influential in her decision about RM. Although both sisters participated in breast screening, their tumours were identified between their regular mammograms. This woman did not want to risk being diagnosed with breast cancer later in life or regret not having done everything she could to prevent it.

At the second follow-up interview three months later, this woman confirmed she had undergone RM, one month earlier. She stated that "even up to the very end I was saying, oh my God, oh my God." She recalled that she re-read or referred to all the sections of the RM booklet in the months preceding surgery. When she accepted the RM surgery date, she described still feeling unsettled about the decision until her husband "cleared the air" for her. He had left the RM decision to her, but when she mentioned to him that she might not go through with the surgery, he told her: "Maybe you *have *to look at this as something you *have *to do for your health." After hearing this, she recalled, "I knew absolutely that day, that the decision I was making was the right decision for me." Reassured that her adult children and surgeons also believed her decision to be a good one for her situation, she had felt ready to go forward with the surgery.

Although she acknowledged that the process had not been easy, she expressed definite satisfaction with the decision after the surgery was complete. Throughout the decision-making process, her decision confidence score remained very high, her decision conflict score remained consistently moderate, her perceived knowledge of RM score steadily increased and her perceived knowledge of breast cancer screening decreased (see Table 1). She described the immediate after-effects of the RM as "amazing," feeling as though "a load was off my mind". She stated that this was the first time since her sister was diagnosed with breast cancer five years earlier that she was able to feel carefree about life.

Although ultimately deciding to have RM, she highlights the difficulties of her decision making in the following way:

It was just the thought of, was I doing something needlessly. And was I being selfish, in wanting to, let's say okay, not me, you know. It was just simply making the decision, that was just, it was just. At first I thought it was pretty easy to say 'Well okay I have some options here now', and one of the ones is I can have prophylactic mastectomy. And I just thought that was a more definite thing, and, uh, the most challenging thing to me was that nobody would tell me, nobody would tell me what to do. Like, I ultimately had to make the decision myself. That was the most challenging I think.

## Discussion

The experiences described in this study represent three different RM decision outcomes by three healthy women who carry a *BRCA1/*2 gene mutation, received the RM information booklet to support their decision making, and were initially leaning towards RM. Case #1 decided against RM; over the course of a year, she clarified her values, gathered information, and changed her initial decision. Case #2 remained undecided about whether to have RM. As she gathered more information over the year, she became more ambivalent about RM. Case #3 proceeded with RM, despite several months of increasing doubt after making that decision. She searched for reassurance and encouragement to proceed with surgery, before confirming her decision.

The women represented by these cases demonstrate three different decision-making processes related to the option of RM and underline the importance of time in that process. Each woman felt she was leaning towards RM when first informed about the surgical option near the time of receiving her genetic test results. Those initial thoughts about RM, however, evolved over the course of follow-up, which ranged from eight to twelve months. For these three women, it appeared important to take time to make an informed decision about RM, one that they could live with comfortably, that reflected their own needs and values, and that was based on a good knowledge of the risks and benefits of surgery and the effectiveness of other cancer risk-reducing strategies. Although the comprehensive, longitudinal data collected in this prospective study provided a rich, detailed description of each woman's experience, which was verified by each participant, we recognize that there may be other RM decision-making experiences that are not represented by this case series.

Women who live with a *BRCA1/2 *gene mutation face a difficult and complex decision regarding RM, and these three cases highlight some of the challenges inherent in this decision. All three women focused strongly on quality of life issues, specifically the risk of decreased quality of life (i.e., associated with the impact of surgery on their physical health, body image and relationships) versus the benefit of a significant reduction in breast cancer risk. Little research exists that addresses the quality of life issues for women who have undergone RM [15]. Struggling with uncertainties, these women spent much time reviewing the study RM booklet as well as locating additional sources of information in an attempt to resolve their own questions about the impact of RM on their lives. In some instances, the uncovering of new information raised new concerns; for example, learning more about breast reconstruction generated questions for Case #2 about the comparative risks and details of the different surgical choices.

Hallowell has observed that health care providers approach discussions with women about RM differently than other cancer risk management choices [16]. The women participating in our study consistently expressed frustration when their interactions with health care providers failed to yield specific recommendations. They wanted a response from health care providers as to the appropriateness of their decision in light of their cancer risk. Women's uncertainty in the decision-making process was heightened when providers indicated that RM is a woman's personal decision. On the other hand, while women looked for medical reinforcement of their decisions, when a health care provider indicated an opinion that was not in line with one woman's beliefs, this situation became an additional source of frustration. Further research is needed about the consequences of health care providers offering advice related to RM while at the same time supporting autonomous decision making.

Few studies in the literature address the nature and characteristics of the RM decision-making process in terms of time or the importance of decision support. Two studies that utilized an intervention, either a decision aid or individual survival curves as part of a decision support system, showed improvement in satisfaction with the decision but did not assess changes in the decision over time [11,12]. In the three cases reported here, initial decisions were reviewed and revised several times over a period of six months to a year before a "final" decision was made. The extent to which this extended decision-making process is a by-product of the health care system in Canada in which lengthy wait times exist for elective surgeries such as RM, or is intrinsic to an irreversible and life-altering surgery decision, is unknown. The paucity of research on psychological consequences of RM and decision-making processes was noted by the authors of a Swedish study of 56 women at high risk for familial breast cancer and considering RM [17]. They reported that the process of reaching a decision about RM took about one year to enable the women to reflect adequately on their options. Interestingly, 91% of the women elected to undergo RM (however 16 of these women had previously been affected with breast cancer). These women decided about RM after they received a collaborative recommendation from a multi-disciplinary team that included geneticists, oncologists, breast surgeon, plastic surgeon, nurses and a psychologist. At 2-year follow-up, the majority of women in this study expressed satisfaction with the procedure. While cultural differences may account the for the high rate of acceptance of RM in the Swedish study, the strong level of integrated decision support stands in contrast to the experiences of women in our study.

In a Dutch study, 51% of unaffected female *BRCA1/2 *gene mutation carriers elected to have RM, with 89% of these women making their decision within nine months of receiving their genetic testing results [18]. These authors focused on the importance of counselling and the benefit of reduced fear in reducing the likelihood of decision regret, and did not address the time women may need to fully weigh the issues involved in RM. Again, it is difficult to separate out any cultural contribution that may account for the Dutch results, however, all three of our cases initially reported being in favour of RM and yet as they became more informed and gathered more information and opinions and about this surgical option, their decisions changed.

High-risk women considering RM as a strategy to reduce breast cancer risk, and thereby reduce fear, have been characterized largely as a homogenous group defined by the results of their genetic test. Other researchers have investigated women with family histories of breast and ovarian cancer using the concept of "chronic risk", to focus on the ongoing adaptation that occurs in the lives of women with heightened cancer risk perception [19,20]. Results suggest that perceptions of heightened risk and associated decisions fluctuate over time, increasing or decreasing, depending on life events and experiences, such as reaching the age when close family members were diagnosed with cancer in the past, friends or family members developing cancer, life stage in terms of child-bearing and child-rearing, and experiences with false positive results of cancer screening tests [20].

A staged or phased model of coming to terms with one's personal perception of risk has been identified and described by Chalmers and Thomson [19]. Their qualitative research with women at high risk for developing breast cancer revealed three interdependent phases that included living the cancer experience, developing a risk perspective, and putting risk in its place. Women were found to integrate the knowledge of being at risk into their self-identity by being either a "controlling woman" or "non-controlling woman". A controlling woman is more likely to make dietary changes and participate in screening and breast self-exam, whereas a non-controlling woman is less likely to adopt lifestyle changes and expresses a more fatalistic view concerning cancer. "Putting risk in its place" did not occur for all women, and was not a static process, indicating that some women are more able to live with the perception of chronic breast cancer risk than others. Press et al [21] argued that RM may have different meanings for women, and they explained the variation in women's uptake of RM in the United States, in terms of the distinction between illness and disease. Women who see RM as mimicking the illness of breast cancer, as opposed to preventing the disease, are less likely to consider the procedure an option. They also point out that because actual uptake of RM is higher in several international studies than any reported hypothetical interest in the surgery, it is important to gain a better understanding of how women perceive RM.

## Conclusion

Given the lack of available research that has examined women's decision-making processes and the psychological and medical outcomes of RM, it may be wise for health care providers to view the decision-making process as fluid and one that may involve extended or recursive processes, dependent on women's psychological coping style, values, life experiences and circumstances. Women with a *BRCA1/2 *gene mutation may choose to revisit the RM decision at different stages of their lives and, therefore, require long-term professional support in a decision-making process that is contextualized by medical uncertainty and a lack of conclusive information. Longitudinal studies that investigate RM decision-making outcomes over time are needed to understand how best to support women in this decision-making process.

## Competing interests

The author(s) declare that they have no competing interests.

## Authors' contributions

MM conceived the study, participated in its design, recruited participants, conducted follow-up consultations, analyzed transcripts and helped draft the manuscript. JLB conceived the study, participated in its design and coordination, analyzed transcripts and helped draft the manuscript. LGB participated in study design and conducted interviews. SAK participated in study design, conducted interviews, performed data analysis and helped prepare the manuscript. MK performed data analysis and drafted the manuscript. All authors read and approved the final manuscript.

## Pre-publication history

The pre-publication history for this paper can be accessed here:



## References

[B1] Ford D, Easton DF, Stratton M, Narod S, Goldgar D, Devilee P, Bishop DT, Weber B, Lenoir G, Chang-Claude J, Sobol H, Teare MD, Struewing J, Arason A, Scherneck S, Peto J, Rebbeck TR, Tonin P, Neuhausen S, Barkardottir R, Eyfjord J, Lynch H, Ponder BA, Gayther SA, Zelada-Hedman M, the Breast Cancer Linkage Consortium (1998). Genetic heterogeneity and penetrance analysis of the BRCA1 and BRCA2 genes in breast cancer families. Am J Hum Genet.

[B2] Meiser B, Butow P, Price M, Bennett B, Berry G, Tucker K (2003). Attitudes to prophylactic surgery and chemoprevention in Australian women at increased risk for breast cancer. Journal of Women's Health.

[B3] Meiser B, Butow P, Friedlander M, Schnieden V, Gattas M, Kirk J, Suthers G, Haan E, Tucker K (2000). Intention to undergo prophylactic bilateral mastectomy in women at increased risk of developing hereditary breast cancer. Journal of Clinical Oncology.

[B4] van Dijk S, Otten W, Zoeteweij MW, Timmermans DRM, van Asperen CJ, Breuning MH, Tollenaar R, Kievit J (2003). Genetic counselling and the intention to undergo prophylactic mastectomy: effects of a breast cancer risk assessment. British Journal of Cancer.

[B5] Stefanek M, Hartmann L, Nelson W (2001). Risk-reduction mastectomy: clinical issues and research needs.. Journal of the National Cancer Institute.

[B6] van Oostrom I, Meijers-Heijboer H, Lodder LN, Duivenvoorden HJ, van Gool AR, Seynaeve C, van der Meer CA, Klijn JGM, van Geel BN, Burger CW, Wladimiroff JW, Tibben A (2003). Long-term psychological impact of carrying a BRCA1/2 mutation and prophylactic surgery: a 5-year follow-up study. Journal of Clinical Oncology.

[B7] Hatcher MB, Fallowfield LJ (2003). A qualitative study looking at the psychosocial implications of bilateral prophylactic mastectomy. The Breast.

[B8] Zion S, Slezak J, Sellers T, Woods J, Arnold PG, Petty P, Donohue J, Frost M, Schaid D, Hartmann L (2003). Reoperations after prophylactic mastectomy with or without implant reconstruction. Cancer.

[B9] Bresser PJC, Seynaeve C, van Gool AR, Brekelmans CT, Meijers-Heijboer H, van Geel AN, Menke-Pluijmers MB, Duivenvoorden HJ, Klijn JGM, Tibben A (2006). Satisfaction with prophylactic mastectomy and breast reconstruction in genetically predisposed women. Plastic and Reconstructive Surgery.

[B10] Metcalfe KA, Semple JL, Narod SA (2004). Satisfaction with breast reconstruction in women with bilateral prophylactic mastectomy: a descriptive study. Plastic and Reconstructive Surgery.

[B11] Armstrong K, Weber B, Ubel PA, Peters N, Holmes J, Schwartz JS (2005). Individual survival curves improve satisfaction with cancer risk management decisions in women with BRCA1/2 mutations. Journal of Clinical Oncology.

[B12] van Roosmalen MS, Stalmeier PFM, Verhoef LCG, Hoekstra-Weebers JEHM, Oosterwijk JC, Hoogerbrugge N, Moog U, van Daal WAJ (2004). Randomised trial of a decision aid and its timing for women being tested for a *BRCA1/2* mutation. British Journal of Cancer.

[B13] Hallowell N (1998). "You don't *want* to lose your ovaries because you think 'I might become a man' ". Women's perceptions of prophylactic surgery as a cancer risk management option. Psycho-oncology.

[B14] Hallowell N, Potts L (2000). Reconstructing the body or reconstructing the woman? Perceptions of prophylactic mastectomy for hereditary breast cancer risk.. Ideologies of Breast Cancer.

[B15] Anderson B (2001). Prophylactic surgery to reduce breast cancer risk: a brief literature review. The Breast Journal.

[B16] Hallowell N (1999). Advising on the management of genetic risk: offering choice or prescribing action?. Health, Risk & Society.

[B17] Brandberg Y, Arver B, Lindblom A, Sandelin K, Wickman M, Hall P (2004). Preoperative psychological reactions and quality of life among women with an increased risk of breast cancer who are considering a prophylactic mastectomy. European Journal of Cancer.

[B18] Meijers-Heijboer EJ, Verhoog LC, Brekelmans CTM, Seynaeve C, Tilanus-Linthorst MMA, Wagner A, Dukel L, Devilee P, van den Ouweland AMW, van Geel AN, Klijn JGM (2000). Presymptomatic DNA testing and prophylactic surgery in families with a BRCA1 or BRCA2 mutation. Lancet.

[B19] Chalmers K, Thomson K (1996). Coming to terms with the risk of breast cancer: perceptions of women with primary relatives with breast cancer. Qualitative Health Research.

[B20] Kenen R, Ardern-Jones A, Eeles R (2003). Living with chronic risk: healthy women with a family history of breast/ovarian cancer. Health, Risk & Society.

[B21] Press N, Reynolds S, Pinsky L, Murthy V, Leo M, Burke W (2005). 'That's like chopping off a finger becasue you're afraid it might get broken': disease and illness in women's views of prophylactic mastectomy. Social Science & Medicine.

[B22] O'Connor AM (1995). Validation of a decisional conflict scale. Medical Decision Making.

[B23] Song M, Sereika S (2006). An evaluation of the Decisional Conflict Scale for measuring the quality of end-of-life decision making. Patient Education and Counseling.

[B24] Bastian LA, McBride C, Fish L, Lyna P, Farrell D, Lipkus IM, Rimer B, Siegler IC (2002). Evaluating participants' use of a hormone replacement therapy decision-making intervention. Patient Education and Counseling.

